# Characterization of Morphology and Composition of Inorganic Fillers in Dental Alginates

**DOI:** 10.1155/2014/178064

**Published:** 2014-07-24

**Authors:** Ricardo Danil Guiraldo, Sandrine Bittencourt Berger, Rafael Leonardo Xediek Consani, Simonides Consani, Rodrigo Varella de Carvalho, Murilo Baena Lopes, Luciana Lira Meneghel, Fabiane Borges da Silva, Mário Alexandre Coelho Sinhoreti

**Affiliations:** ^1^Department of Restorative Dentistry, School of Dentistry, University North of Parana (UNOPAR), Rua Marselha 183, 86041-140 Londrina, PR, Brazil; ^2^Department of Prosthodontics and Periodontics, Piracicaba Dental School, State University of Campinas (UNICAMP), Avenida Limeira 901, 13414-903 Piracicaba, SP, Brazil; ^3^Department of Restorative Dentistry, Piracicaba Dental School, State University of Campinas (UNICAMP), Avenida Limeira 901, 13414-903 Piracicaba, SP, Brazil

## Abstract

Energy dispersive X-ray spectroscopy microanalysis (EDX), scanning electron microscopy (SEM), and Archimedes' Principle were used to determine the characteristics of inorganic filler particles in five dental alginates, including Cavex ColorChange (C), Hydrogum 5 (H5), Hydrogum (H), Orthoprint (O), and Jeltrate Plus (JP). The different alginate powders (0.5 mg) were fixed on plastic stubs (*n* = 5) and sputter coated with carbon for EDX analysis, then coated with gold, and observed using SEM. Volume fractions were determined by weighing a sample of each material in water before and after calcining at 450^°^C for 3 h. The alginate materials were mainly composed of silicon (Si) by weight (C—81.59%, H—79.89%, O—78.87%, H5—77.95%, JP—66.88%, wt). The filler fractions in volume (vt) were as follows: H5—84.85%, JP—74.76%, H—70.03%, O—68.31%, and C—56.10%. The tested materials demonstrated important differences in the inorganic elemental composition, filler fraction, and particle morphology.

## 1. Introduction

Alginate impression materials are commonly used for making diagnostic and working casts due to their ease of use, low cost [1], and good patient acceptance [2]. Many factors influence the ultimate success of prostheses, including the setting characteristics [2, 3], the rheological properties after setting [4], and compatibility with dental stones [1, 5]. Alginates are a two-component molding system in which a powdered material is mixed with water. The powder contains sodium or potassium alginate (soluble alginate), a diatomaceous earth filler, calcium sulfate as a reactant, fluoride as an accelerator, and sodium phosphate as a retarder [6]. Excellent surface detail reproduction and dimensional accuracy are necessary to produce a true copy of an anatomical structure, and these properties are commonly used to analyze the performance of impression materials [7].

Although the hydrophilic nature of irreversible hydrocolloids is valuable for making impressions in moist environments, this characteristic also limits their use. Irreversible hydrocolloids are affected by syneresis and imbibition, and stone casts must therefore be fabricated as soon as possible to avoid dimensional changes. The effects of storage on the dimensional accuracy and deformation of gypsum casts formed from alginate impressions have previously been described, with one study reporting that dimensional changes in alginate impressions varied between brands [8]. Impressions are generally filled with dental gypsum as quickly as possible to avoid long exposure to air and the resulting syneresis and evaporation. If immediate pouring is not possible, it is recommended that the impression be kept in a 100% relative humidity environment to preserve the water balance within the material. Alginate manufacturers typically recommend that models be poured within 12 hours because an increased dimensional change occurs after 12–24 hours [9]. It was found that storage for up to 3 hours after the impressions were sprayed with the disinfectant resulted in less than 24 *μ*m change; therefore, no deformation was observed in the casts [10]. However, manufacturers (Cavex Holland BV and Zhermack) claim that their alginates (Cavex ColorChange and Hydrogum 5, resp.) may remain stable for 5 days without any change in their properties.

Therefore, the purpose of this study was to investigate whether there are differences in the inorganic composition of filler particles in several dental alginate formulations by energy dispersive X-ray spectroscopy microanalysis (EDX). In addition, the filler particles morphology/size were determined using scanning electron microscopy (SEM) and the filler fraction of commercial alginates was investigated by Archimedes' Principle. The null hypotheses tested were that there is no difference in (1) composition, (2) filler particle morphology/size, or (3) filler content among dental alginate materials.

## 2. Materials and Methods

The alginate impression materials Cavex ColorChange (batch number 100221, Cavex Holland BV, Haartem, The Netherlands), Hydrogum 5 (batch number C302070, Zhermack, Badia Polesine, RO, Italy), Hydrogum (batch number 116304, Zhermack, Badia Polesine, RO, Italy), Orthoprint (batch number 118190, Zhermack, Badia Polesine, RO, Italy), and Jeltrate Plus (batch number 420010C, Dentsply Caulk, Milford, DE, USA) were used in this study.

### 2.1. Inorganic Composition

Alginate powder amounts of 0.5 mg were used from each material (*n* = 5), according to the previous study [6]. The alginate powders were fixed in plastic stubs, sputter coated with carbon (MED 010, Balzers, Balzer, Liechtenstein) to eliminate the charging effects. After that, the samples were observed by scanning electron microscope (SEM)/energy dispersive X-ray analysis (EDX).

The EDX was used to detect the main inorganic components of the tested materials. Specimens were identified by using a SEM operating with a Vantage System (Noran Instruments, Middleton, WI). The spectra for EDX measurements were obtained for 100 s livetime (voltage: 15 kV; dead time 20–25%; working distance: 20 mm) [11].

### 2.2. Filler Morphology and Size

Following the EDX analysis, the samples were coated with gold/palladium under high vacuum (SCD 050, Bal-tec AG, Liechtenstein) and placed in a JSM5600 SEM system (JEOL Ltd., Tokyo, Japan). Images of the filler particles in each alginate material were obtained at 1200x magnification (voltage: 15 kV; beam width: 25–30 nm; working distance: 10–15 mm) [11]. The SEM images were imported and analyzed using an image-analysis system (ImageJ 1.41; Wayne Rasband, National of Institutes of Health, Bethesda, MD, USA). At least 20 particles of each material were analyzed during this procedure to determine the maximum, minimum, and mean particle diameter in micrometers (*μ*m).

### 2.3. Volumetric Filler Fraction

The percentage of inorganic particles by volume was determined by calculating the difference between the mass of each material tested in air and in water (Archimedes' Principle) [12]. The materials were manipulated according to the manufacturers' instructions and placed in an aluminum matrix to produce cylindrical specimens (12 mm diameter, 20 mm high) of each material. These specimens were weighed in an analytical balance (JK 180, Chyo Balance Corp., Tokyo, Japan) with an accuracy of 0.0001 g (*n* = 5), according to a previous study [6]. The dried mass (Md) of the material after the setting time was determined in the air. To determine the wet mass (Mi), a recipient and a stainless steel mesh were placed over the balance plate and filled with distilled water, and the specimen was immersed. The volume of the specimen after the setting time was measured according to the following Equation (1) [6]:

(1) $$\begin{array}{c} \text{Vs}=\text{Md}-\text{Mi}. \end{array}$$

The specimens were then calcined in an oven (Bravac Ltda, Sao Paulo, SP, Brazil) to remove the organic constituents. The temperature was gradually increased from room temperature for 1.5 hours to reach 450°C and maintained at this temperature for 3 hours [6]. The remaining inorganic material was intact and pill shaped. The mass in air (Mp) was then measured as described above. To determine the wet mass of the particles (Mpi), the specimens were immersed in distilled water as described above, and at this time, the pill shape was disarranged because of its contact with the water. The volume of the inorganic particles was measured according to the following equation [6]:

(2) $$\begin{array}{c} \text{Vp}=\text{Mp}-\text{Mpi}. \end{array}$$

The volume percentage of the inorganic phase was calculated using the following [6]:

(3) $$\begin{array}{c} \text{Inorganic}\;\text{particle}\;\text{percentage}=\left(\frac{\text{Vp}}{\text{Vs}}\right)\times 100. \end{array}$$

## 3. Results

### 3.1. Inorganic Composition

**Figure 1:**
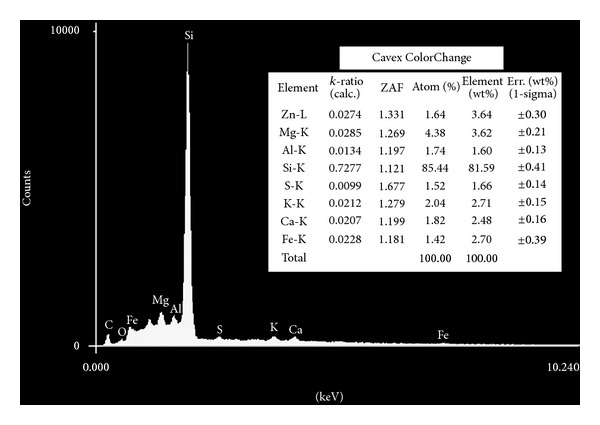
Elements identified by energy dispersive X-ray spectroscopy microanalysis for Cavex ColorChange.

**Figure 2:**
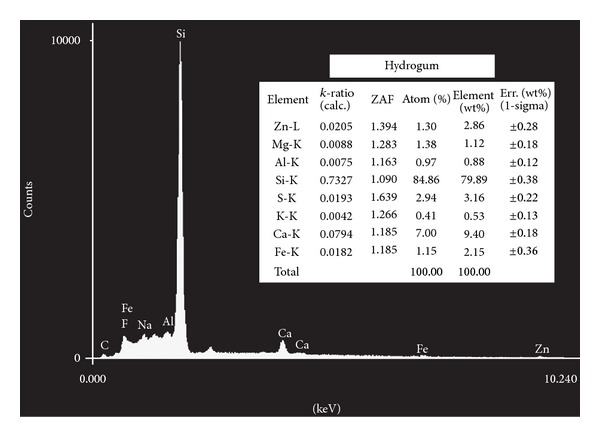
Elements identified by energy dispersive X-ray spectroscopy microanalysis for Hydrogum.

**Figure 3:**
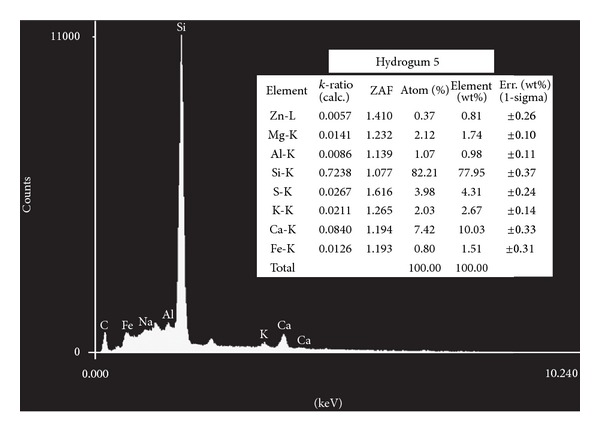
Elements identified by energy dispersive X-ray spectroscopy microanalysis for Hydrogum 5.

**Figure 4:**
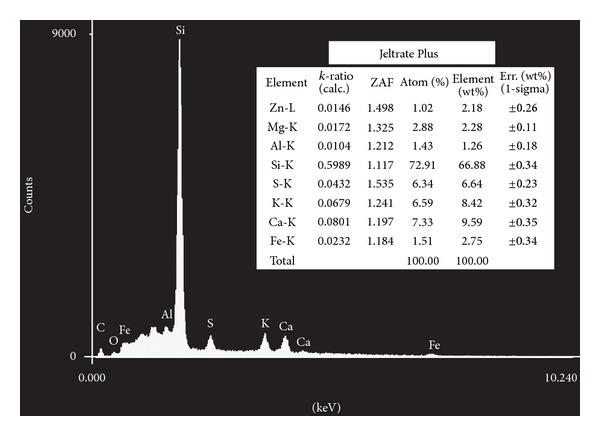
Elements identified by energy dispersive X-ray spectroscopy microanalysis for Jeltrate Plus.

**Figure 5:**
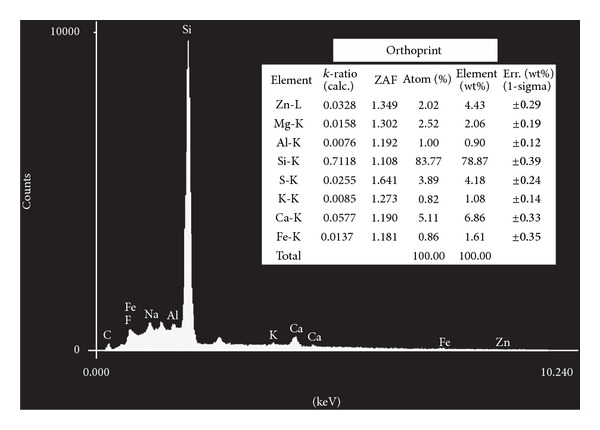
Elements identified by energy dispersive X-ray spectroscopy microanalysis for Orthoprint.

The elements identified using energy dispersive X-ray microanalysis appear in Figures 1, 2, 3, 4, and 5. Silicon (Si) was the main component by weight in all of the formulations (Cavex ColorChange—81.59% wt, Hydrogum—79.89% wt, Orthoprint—78.87% wt, Hydrogum 5—77.95% wt, Jeltrate Plus—66.88% wt). The remaining components are described in Figures 1–5.

### 3.2. Filler Morphology and Size

**Figure 6:**
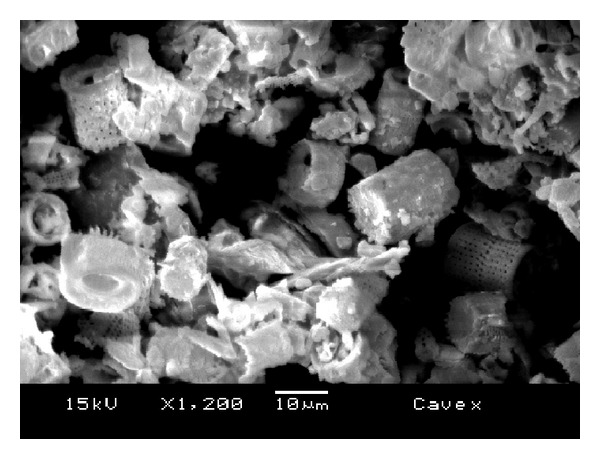
SEM micrograph of Cavex ColorChange alginate impression material; original magnification 1.200x.

**Figure 7:**
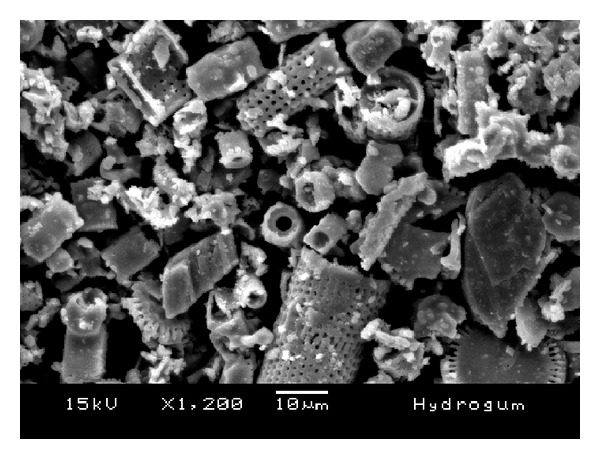
SEM micrograph of Hydrogum alginate impression material; original magnification 1.200x.

**Figure 8:**
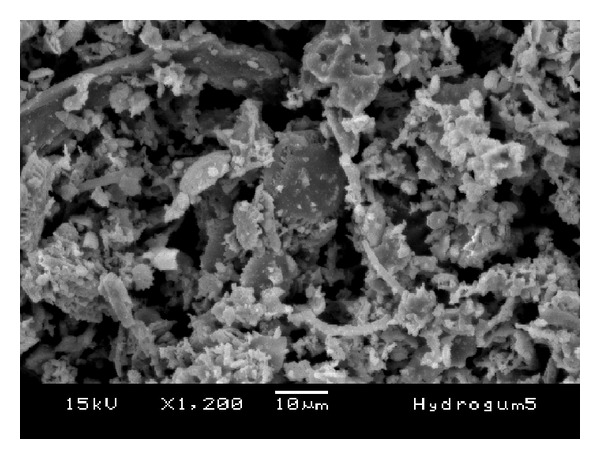
SEM micrograph of Hydrogum 5 alginate impression material; original magnification 1.200x.

**Figure 9:**
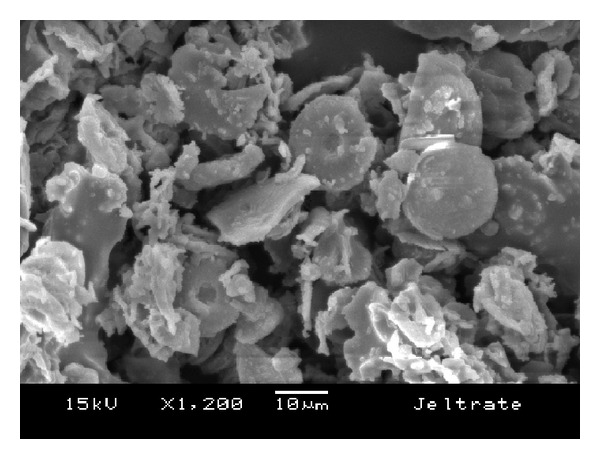
SEM micrograph of Jeltrate Plus alginate impression material; original magnification 1.200x.

**Figure 10:**
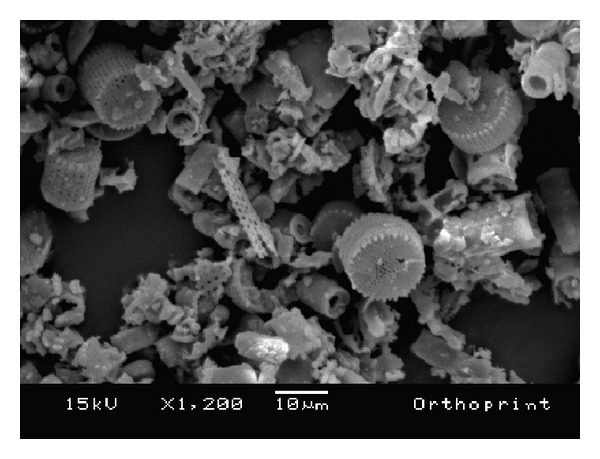
SEM micrograph of Orthoprint alginate impression material; original magnification 1.200x.

The morphology of the fillers is shown in the SEM images in Figures 6, 7, 8, 9, and 10. The inorganic particles of the tested materials showed several shapes and sizes. The Hydrogum 5 and Jeltrate Plus materials showed circular and helical particles with several perforations. The Hydrogum, Cavex ColorChange, and Ortoprint materials showed particles with cylindrical and perforated sticks' shapes.

The maximum, minimum, and mean diameter size values of the inorganic particles are listed in Table 1. Jeltrate Plus showed the highest mean values for diameter size. Because of the difference of Cavex ColorChange, Hydrogum, and Orthoprint particle shapes, which had a considerable length to be measured, Table 1 presents its maximum, minimum, and mean length values beyond the values for diameter.

### 3.3. Volumetric Filler Fraction

The mean values of the percentage content of inorganic particles in volume are listed in Table 2. Hydrogum 5 presented the highest mean values (84.85% vt), while Cavex ColorChange presented the lowest values (56.10% vt). Jeltrate Plus, Hydrogum, and Orthoprint showed 74.76% vt and 70.03% vt and 68.31% vt, respectively.

## 4. Discussion

The inorganic composition of the alginate materials is described in Figures 1–5. In the past, lead salts were used to replace calcium to enhance the gel through the formation of lead alginate [9]. However, lead is not essential to the formulation of high-quality alginates. de Freitas [13] analyzed the Pb content of 25 dental alginate powders, 20 of which contained Pb in small amounts varying from 0.0007 to 0.095% wt. The materials analyzed in this study did not contain lead, which is considered undesirable due to its toxicity. Although the exposure risk to the dental patient is minimal, even with alginates containing relatively high Pb concentrations [9], there may be some risks associated with the inadvertent ingestion of the alginates or the inhalation of alginate powder during preparation [9].

Alginate powders typically contain sodium or potassium (most commonly found in approximately 15% wt) alginates (soluble alginates), diatomaceous earth (approximately 60% wt) acting as filler particles (consisting of silicon, aluminum, iron, calcium, sodium, magnesium, titanium, and potassium), zinc oxide (approximately 4% wt) acting as filler particles, calcium sulfate (approximately 16% wt) as a reactant, a fluoride (approximately 2% wt) as an accelerator, and sodium phosphate (approximately 2% wt) as a retarder [6, 14]. The main constituent of diatomaceous earth is silica (silicon dioxide) in weight percentages ranging from 58 to 91% wt with more than 12,000 different species. However, in the present study, the silicon content of 5 products varied from 81.59% to 66.88% wt in the EDX analysis. Thus, the current study suggests that this percentage is higher in alginates studied. Furthermore, two other secondary chemical elements of diatomaceous earth (aluminum oxide and iron oxide) did not show high percentages in weight (from 1.51 to 2.75% wt for iron element and from 0.88 to 1.60% wt for the aluminum element). For this reason, the filler loading was calculated using the Archimedes' Principle. Zinc concentrations (filler particles) ranged from 0.81 to 4.43% wt, with Cavex ColorChange and Orthoprint, having the highest values of 3.64 and 4.43% wt, respectively. The MgO presence provides a material with higher tear strength and hardness and a smaller setting time, thus indicating its very important role [15]. Magnesium concentrations ranged from 1.38 to 4.38 wt, with the highest values for Cavex ColorChange. It would be expected that this alginate impression material show improvements in these properties.

The findings of this investigation showed Cavex ColorChange as the material with the lowest results for the volumetric filler fraction (56.10% vt), while Hydrogum 5 had the highest values (84.85% vt). Thus, it is expected that the decrease of soluble alginate on Hydrogum 5 will cause a lower alteration in stability because a lower weight percentage gel is invariably subject to fewer changes in dimension by syneresis, evaporation, and imbibition of water. Differences among materials are not directly related to filler content, but it seems to be very important to be considered [6]. This fact was observed in the study of the Sedda et al. [16], in which the accuracy of casts made with five alginates (different from those used in this study) were assessed immediately and afterward poured with different storage periods, and only the Hydrogum 5 remained stable after a period of 120 hours. On the other hand, materials with a higher percentage of inorganic filler particles may be less susceptible to degradation by disinfection. However, Guiraldo et al. [17] observed no differences in dimensional accuracy when testing various combinations of disinfectant procedures (2% sodium hypochlorite, 2% chlorhexidine digluconate, or 0.2% peracetic acid) and alginate impression materials (Cavex ColorChange, Hydrogum 5, or Jeltrate Plus), possibly because of the disinfection method (spraying) or short contact time (15 minutes).

The inorganic filler particles observed by SEM pictures appear to be the cell walls of algae from the division Chrysophyta, class Bacillariophyceae. The members of this class, referred to as diatoms, are essentially unicellular, although chains of cells and colonial aggregations may occur [6]. There are records of these algae dating from the Cretaceous period. The classification of diatoms is almost entirely based on the structure and ornamentation of the cell wall, which is termed the frustule [6]. Due to their siliceous nature and resistance to natural degradation, the frustules accumulate in geological layers within the earth's crust, eventually forming significant deposits. Known as diatomaceous earth, or diatomite, this material is mined and used for a variety of commercial purposes [6].

When added in proper amounts, diatomaceous earth can improve the strength and stiffness of the alginate gel; produce a smooth texture; and ensure a firm, tack-free gel surface [6]. It also aids in dispersing the alginate powder particles in water. Without filler, alginate gels lack firmness and possess a sticky surface covered with an exudate produced as a result of syneresis [14]. The size and amount of filler and gel affect the accuracy of the alginate impression [14]. Because of this, Orthoprint and Cavex ColorChange should provide the best detail reproduction because of their lower mean diameters (Orthoprint—7.94 *μ*m) and low filler fraction (Cavex ColorChange—56%). However, this was not observed by Guiraldo et al. [17] when testing three brands of alginate (Cavex ColorChange, Hydrogum 5, and Jeltrate Plus) possibly because the accuracy was evaluated solely on the basis of reproduction of a 50 *μ*m line in accordance with the ISO standard. The comparisons are also hindered by the fact that the fillers in Hydrogum 5 and Jeltrate Plus exhibit a morphology different from the other materials, resembling colonial aggregates from a different order, suborder, or genus.

Moreover, the ideal properties of impression materials are as follows: be fluid to reproduce details with accuracy; have sufficient viscosity to stay in tray; set in the oral environment in a short period of time, up to 7 minutes; do not distort after set; have dimensional stability until pouring; have the possibility of pouring more than once; be biocompatible; and do not tear during removal from the mouth. Thus, alginates should meet the maximum of these requisites. The stiffness and strength are directly related to the filler concentration of hydrocolloid [14]. The strength of reversible (alginates') gels can be increased by the addition of fillers [14]. Thus, clinically, alginates with higher filler concentrations could have higher tear strength. In the present study, Hydrogum 5 showed better values of filler concentration and could show better tear strength. However, this fact is a limitation of this study because the tear strength was not assessed, and further studies are needed to confirm these results. Therefore, based on the obtained results, the null hypotheses were not accepted, as there were differences in (1) composition, (2) filler particle morphology/size, or (3) filler content among dental alginate materials.

## 5. Conclusions

Based on the results from our study and within its limitations, the tested materials demonstrated important differences in the inorganic elemental composition, filler fraction, and particle morphology. These differences can predict the mechanical properties and clinical outcome of these alginates.

## Figures and Tables

**Table 1:** Maximum, minimum, and mean values for alginate filer size (*µ*m).

Material	Maximum	Minimum	Mean
Cavex ColorChange (diameter)	16.32	6.48	11.16
Cavex ColorChange (length)	20.16	6.63	12.79
Hydrogum 5 (diameter)	28.64	4.02	11.43
Hydrogum (diameter)	24.23	4.43	8.52
Hydrogum (length)	29.02	4.82	13.16
Orthoprint (diameter)	18.82	4.04	7.94
Jeltrate Plus (diameter)	24.18	7.83	13.07

**Table 2:** Mean values for volumetric filler fraction of alginates (%).

Material	Volumetric filler fraction
Hydrogum 5	84.85
Jeltrate Plus	74.76
Hydrogum	70.03
Orthoprint	68.31
Cavex ColorChange	56.10
